# Neuroimaging-based pain biomarkers: definitions, clinical and research applications, and evaluation frameworks to achieve personalized pain medicine

**DOI:** 10.1097/PR9.0000000000000762

**Published:** 2019-08-07

**Authors:** Sean Mackey, Henry T. Greely, Katherine T. Martucci

**Affiliations:** a Division of Pain Medicine, Department of Anesthesiology, Perioperative and Pain Medicine, Stanford University School of Medicine, Palo Alto, CA, USA; b Director of the Stanford Program in Neuroscience and Society, Stanford Law School, Center for Law and the Biosciences, Stanford, CA, USA; c Department of Anesthesiology, Duke University School of Medicine, Durham, NC, USA

**Keywords:** Neuroimaging, MRI, Classification, Prognosis, Prediction, Biomarkers, Chronic pain, Signature, Pattern, Multivariate

## Abstract

One of the key ambitions of neuroimaging-based pain biomarker research is to augment patient and clinician reporting of clinically relevant phenomena with neural measures for prediction, prognosis, and detection of pain. Despite years of productive research on the neuroimaging of pain, such applications have seen little advancement. However, recent developments in identifying brain-based biomarkers of pain through advances in technology and multivariate pattern analysis provide some optimism. Here, we (1) define and review the different types of potential neuroimaging-based biomarkers, their clinical and research applications, and their limitations and (2) describe frameworks for evaluation of pain biomarkers used in other fields (eg, genetics, cancer, cardiovascular disease, immune system disorders, and rare diseases) to achieve broad clinical and research utility and minimize the risks of misapplication of this emerging technology. To conclude, we discuss future directions for neuroimaging-based biomarker research to achieve the goal of personalized pain medicine.

## 1. Introduction

Magnetic resonance imaging (MRI) has opened a window to the brain by allowing noninvasive study of both structure and function. Neuroimaging has elucidated how pain processing is linked within the central nervous system (CNS); how it is disrupted in chronic pain; and how those disruptions occur with the chronification of pain.^18,20,62,63,67,75,108^ However, to date, functional MRI (fMRI) has provided minimal direct clinical application for pain.

Identifying and validating neuroimaging-based biomarkers and surrogate endpoints for pain would be useful for clinical and research communities in several ways: (1) prognosis (ie, for indicating the likely progression of pain after injury or surgery,^5,74^ or the progression from chronic pain to high-impact chronic pain^57,58,102^), (2) identifying likely patient responders to a particular treatment (ie, prediction), (3) identifying a specific pain disorder (ie, diagnosis), (4) identifying targets for therapeutic intervention, and (5) defining surrogate endpoints to augment clinical endpoints and predict clinical benefit (N.B. Food and Drug Administration [FDA] validation of surrogate endpoints requires a different validation process than biomarkers, but are listed here in the context of biomarkers). Valid neuroimaging-based biomarkers of pain would also be useful in providing evidence in the legal system. The benefits and limitations of machine learning and classification techniques that can assess neuroimaging data for meaningful patterns of neural structure and function have been reviewed elsewhere.^86^ Preliminary brain biomarkers have been identified in individuals experiencing acute and chronic pain.^3,11,12,16,50,51,56,61,103,110,111,114^ Potential future applications of this technology are exciting; however, the field of brain biomarkers of pain is still in its early phases. As such, we should not prematurely apply neuroimaging biomarkers of pain generally for clinical or legal purposes, until proper validation is performed. Although the task of formal validation is large, we can learn from other fields how to systematically approach the validation of neuroimaging pain biomarkers. Finally, as the field evolves, it is important to consider ethical, social, and legal implications of future validated biomarkers and how or whether they should be used, as previously reviewed.^19,21,35^

Our goals of this article are to (1) review the different types of potential neuroimaging-based biomarkers, their clinical and research applications, and limitations and (2) describe frameworks used in other fields (eg, genetics, cancer, cardiovascular disease, immune system disorders, and rare diseases) for the future validation of pain biomarkers to achieve broad clinical and research utility and minimize the risks of misapplication of this emerging technology. Although we focus predominantly on MRI-based pain biomarkers, there are a variety of imaging methods to characterize structure (eg, quantitative morphometry, white matter “connectivity,” gray matter ultrastructure, and cytoarchitectonic mapping) and function (eg, physiology [cerebral blood flow], metabolism, receptor distribution, gene and protein expression, electrophysiology, and functional connectivity). Of note, the purpose of this article is not to provide a comprehensive review of brain imaging in clinical and experimental pain or of brain-based biomarkers for pain. For those purposes, there are several recent comprehensive reviews.^19,63,64,72,73,98,109,112^

## 2. The BEST way forward in developing neuroimaging biomarkers for pain: definitions, applications, and utility

Pain is a complex physiological and psychological experience,^70^ making it both subjective and inherently difficult to study and treat. Individual variability in pain perception poses additional challenges to assessing and treating pain.^17,29,76^ The most common means to determine whether an individual has pain in both research and clinical settings is subjective reporting. For example, rating pain on a scale from 0 to 10 is an extensively validated and useful measure.^79^ However, in some clinical situations, individuals are unable to report their pain, such as in very young, elderly, infirmed, and unconscious patients. In these cases, objective biomarkers of pain could be helpful.

**Figure 1.**
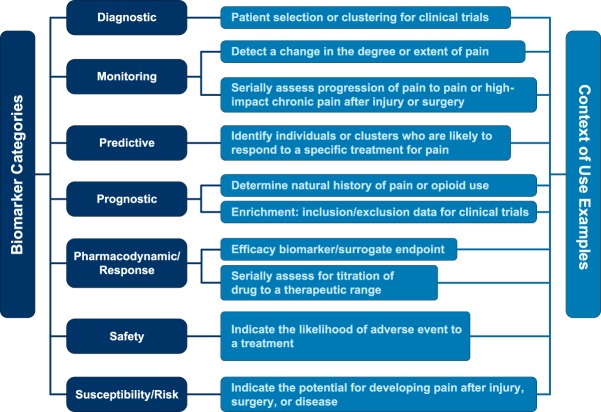
Biomarker Definitions with Context of Use Examples. Adapted from the 2016 FDA-NIH Biomarker Working Group glossary, BEST (Biomarkers, EndpointS, and other Tools Resource)^1^. FDA, Food and Drug Administration.

It is of importance to first clearly and precisely define the types of biomarkers to assure effective and unambiguous communication. The 2016 FDA-NIH Biomarker Working Group glossary, BEST (Biomarkers, EndpointS, and other Tools Resource),^7^ defines a biomarker *as “a characteristic that is objectively measured and evaluated as an indicator of normal biological processes, pathogenic processes, or pharmacologic responses to a therapeutic intervention.”* Thus, a biomarker is not an assessment of how an individual feels, functions, or survives—the characteristics of which more appropriately defines a *clinical endpoint*. The BEST glossary also provides a framework for conceptualizing the multiple types and uses of biomarkers. We draw heavily from the BEST Working Group and provide several of these categories of biomarkers and their potential application to clinical, research, and legal aspects of pain below. Although we focus on neuroimaging biomarkers of pain in this review, the concepts apply to other potential biomarkers of pain (eg, molecular, histologic, radiographic, or physiologic). Figure 1 summarizes the biomarker definitions.

### 2.1. Diagnostic biomarkers

This type of biomarker detects or confirms the presence of a condition or identifies individuals within a specific subtype of a condition.^7^ Pertinent to neuroimaging-based pain biomarkers, the research field has primarily focused on developing diagnostic biomarkers. Our group's initial effort was to address a simple question of whether a pattern or signature of brain activity identified in a training set could be used to accurately determine whether individuals (not part of the training set) were experiencing pain to a thermal stimulus.^12^ For this goal, we trained a linear support vector machine learning algorithm to distinguish painful from nonpainful stimuli with more than 80% classification accuracy. Subsequently, Wager et al.^103^ demonstrated the ability of using a trained brain signature to distinguish between brain states experiencing painful heat and nonpainful warmth; pain anticipation and pain recall; physical and social pain; physical pain and the empathy of pain^49^; and the presence or absence of pain self-regulation.^110^ Other groups have extended these general concepts and methodology to distinguish the presence or absence of various chronic pain states, including but not limited to chronic low back pain,^100^ fibromyalgia,^56^ irritable bowel syndrome,^53^ pelvic pain,^3,50^ and trigeminal neuralgia^114^; further details can be found in the following reviews.^73,109^

Along with a rapid growth in the number of research studies focused on neuroimaging-based pain detection, the field has sparked much controversy. Researchers have debated whether chronic pain is a disease of the brain,^95^ whether brain-derived diagnostics (ie, identifiers of disease) of chronic pain are plausible or would be beneficial,^84^ and whether neuroimaging surrogate measures of pain are useful.^59,85^ Some argue that “brain imaging adds neuroanatomical and neurophysiological information, not validity, to pain reports”^95^ and question whether “these biomarkers “mark” the pain itself or just the neural causes and correlates of pain?”^85^ One review^95^ argues that neuroimaging findings merely reflect one part of a chronic pain condition, which affects the person as a whole (across many body systems and functions, both physiologically and psychologically). Another concern for the use of neuroimaging-based pain biomarkers is the concept of reverse inference in that brain biomarkers may not take into account how selectively an area is activated by the mental process in question (eg, pain).^77^ Based on these valid concerns, neuroimaging findings should not and cannot be expected to represent the sole source, cause, or experience of pain. Furthermore, we should be cautious in generalizing findings until there are appropriately well-controlled replication studies.

In addition, researchers, clinicians, and ethicists have raised the issue of potentially detrimental outcomes from false negatives leading people truly suffering from pain to be unjustifiably denied treatment or compensation. Similarly, a false positive could subject a person to unnecessary and risky treatments. There is also the concern of false-negative findings compromising doctor–patient, employee–patient, or family–patient trust.^21^

These concerns are understandable. However, the goal of neuroimaging biomarkers of pain should not be to replace patient self-reporting, but rather to supplement the information from self-reporting. In other words, we do not normally need an MRI to tell us a patient is in pain when we can just ask them. Although the initial studies of diagnostic brain biomarkers of pain were conceptually simple (but methodologically complex), they were necessary to establish the research infrastructure and methods upon which this technology could be expanded to provide true clinical and research utility. We provide some examples of the utility of diagnostic pain biomarkers below.

The practice of pain medicine requires accurate diagnosis of specific pain conditions. Diagnostic biomarkers can help clinicians determine whether a patient has a particular medical condition for which a treatment may be indicated. For example, imagine a 44-year-old woman who falls and injures her wrist. She subsequently develops burning pain in her hand and wrist with swelling and color changes. This presents a clinical challenge to distinguish whether such a presentation is complex regional pain syndrome (CRPS)—a terribly disabling neuropathic pain condition—or simply a delay in healing after injury, as injury itself leads to similar signs and symptoms as CRPS. A brain biomarker to detect the presence or absence of CRPS could be clinically useful to target appropriate treatment in those with CRPS and avoid overtreatment in those without.

As is becoming increasingly appreciated, many chronic pain conditions have subtypes or clusters with markedly different prognoses or responses to a specific treatment. Researchers can use diagnostic biomarkers in clinical trials evaluating chronic pain subtypes to select patients more likely to respond to a specific treatment (ie, to ultimately serve as a predictive biomarker). Finally, the brain regions and networks identified by these biomarkers can serve as therapeutic targets for pharmaceuticals, mind–body interventions, transcranial magnetic stimulation, or deep brain stimulation. For example, Kutch et al. pooled data (n = 1079) across 7 academic sites as part of the NIH Multidisciplinary Approach to the Study of Chronic Pelvic Pain Research Network^54^ to compare those with urological chronic pelvic pain syndrome (UCPPS) with pain-free controls and individuals with fibromyalgia. A subset of individuals (n = 182) underwent fMRI and structural MRI. Individuals with UCPPS reported pain ranging from localized (pelvic) to widespread (throughout the body). The authors found that individuals with widespread UCPPS had increased brain gray matter volume and functional connectivity involving sensorimotor and insular cortices.^50^ These results indicate that individuals with localized UCPPS may represent a different phenotype than those with more widespread pain and may respond differentially to treatment. Development of a brain-based biomarker of pain to better classify subtypes of pain may allow for more efficacious targeting of specific treatments.

For diagnostic brain-based biomarkers to have true clinical and research utility, we first need to assess their performance. A perfect diagnostic biomarker test would detect 100% of all patients with a disease or disease subset (ie, 100% sensitivity for individuals with the disease who test positive) and detect 100% of patients who do not have the disease (ie, 100% specificity for people without the disease who test negative). However, no biomarker test has perfect sensitivity and specificity, thereby requiring tradeoffs among these features. Additional measures of diagnostic biomarker performance include positive predictive value (ie, the proportion of those who tested positive who have the disease or condition) and negative predictive value (ie, the proportion of those who tested negative who do not have the disease or condition).^7^ The diagnostic biomarker must have appropriate analytical validity (ie, the performance of the detection measure itself). For instance, analytical validity must address: (1) the dynamic range of the detection method (will it pick up a positive signal within the range of variability), (2) the precision of the detection method within the full range of the population of interest, and (3) the accuracy of the detection method. This can be a complex problem for imaging biomarkers and especially for signatures which use an algorithm as the actual detection method. Finally, the biomarker must be tested and validated in large samples of the population to which it is intended to be used to assure generalizability. This emphasizes the importance of incorporating base rates or disease prevalence in assessing the generalized performance of a diagnostic biomarker for broad clinical or research purposes.

The concepts of applying epidemiologic base rates, or disease prevalence, to assess the validity of a diagnostic test in a general population are well-founded applications of Bayes theorem. In fact, over 60 years ago, Meehl and Rosen^69^ described how base rates impact the diagnostic utility of a test, noting that utility is strongly influenced by the base rate of the diagnosis in the population of interest.^91^ Classic epidemiologic studies have demonstrated that, contrary to intuition, tests with more than 90% sensitivity and specificity can perform poorly overall when the base rate of the condition is low—a phenomenon known as the “base rate fallacy.”^82^ This issue was raised in an editorial by Robinson et al.,^83^ in which the authors critique multiple brain imaging studies' results for not including base rates. However, in this stage of research discovery, the use of base rates is premature and should be saved for studies that assess clinical validity in a general population (as described below in the section on ACCE), rather than be used in carefully controlled laboratory environments. The following study illustrates these points. In Ung et al.,^100^ we applied machine learning techniques to distinguish individuals with chronic low back pain vs carefully matched healthy controls, and then defined the neural correlates responsible for this distinction. The subjects chosen with low back pain were narrowly screened to have little to no significant emotional distress, no current medications, no radicular symptoms, and no other sites of pain. The advantage of this narrow selection was to reduce confounds and allow for scientific discovery. In other words, the application of epidemiologic base rates would have been inappropriate for the purposes of this and other similar studies seeking to develop and refine methods and techniques. It is important to note that these subjects with low back pain bear little resemblance to real-world patients with chronic low back pain seen in a clinical setting, limiting the generalizability of the results. Fortunately, the field is making rapid advances that will soon allow diagnostic brain-based biomarkers to be tested in the general population. Until then, application of these diagnostic biomarkers for clinical, commercial, or legal applications is premature.^19^

### 2.2. Prognostic biomarkers

Prognostic biomarkers can indicate an increased (or decreased) likelihood of a future clinical event, disease recurrence, exacerbation of a painful condition, or progression in patients with pain.^7^ Neuroimaging-based prognostic biomarkers of pain hold great potential in identifying patients likely to develop persistent pain or opioid use after injury. For example, researchers have investigated the use of resting state functional connectivity after an acute back pain injury to predict persistence of pain.^5,42^ These authors noted that when pain persisted, brain gray matter density decreased; in addition, greater functional connectivity of the nucleus accumbens with prefrontal cortex predicted pain persistence.^5,42^

Prognostic biomarkers could also be useful in defining the natural history of a painful condition or disease progression. For example, as part of a multicenter clinical trial to characterize chronic pelvic pain, Kutch et al.^51^ used resting state functional connectivity to significantly predict short-term (3-month) pain reduction in individuals with chronic pelvic pain with 73.1% accuracy (69.2% sensitivity and 75.0% precision). In addition, prognostic biomarkers could aid in targeted clinical trials by selecting patients more likely to have pain conditions at high risk of exacerbation and therefore thought more likely to respond to a particular treatment.

### 2.3. Susceptibility/risk biomarker

A susceptibility/risk biomarker is one associated with an increased, or decreased, risk of developing a chronic pain condition in an individual who does not yet have that condition. This contrasts with prognostic biomarkers noted above, which are used to indicate an increased or decreased likelihood of a clinical event in an individual who *already has the painful condition*.

At the time of this writing, we were unable to identify any published research involving neuroimaging to track healthy people without pain and use brain biomarkers to predict who will develop chronic pain. The NIH is planning on funding large scale multicenter trials to identify such susceptibility/risk biomarkers in musculoskeletal pain and after surgery. These trials will include neuroimaging, and the results should yield valuable biomarkers to identify who is vulnerable to the development of chronic pain. Here, we will use examples from other non-neuroimaging fields to illustrate the utility of such biomarkers in predicting risk of developing chronic pain after surgery, injury, disease, or idiopathically.

We and other researchers have identified preoperative risk factors for the development of persistent pain and opioid use after surgery.^14,38,40,41^ Recently, Hah et al.^39^ used a k-means clustering approach in 422 patients undergoing surgery to identify risk predictors of who would develop persistence of pain and opioid use and delayed recovery postoperatively. They identified a possible uniform predictor of disparate surgical outcomes long after hospital discharge. With accurate neuroimaging susceptibility/risk biomarkers, clinical trials could test therapeutic interventions in those most likely to develop persistent pain or opioid use, thereby sparing those at less risk from potentially untoward adverse events from unnecessary intervention.

The Orofacial Pain: Prospective Evaluation and Risk Assessment (OPPERA) study was an NIH-funded, multicenter, cross-disciplinary investigation of the development of temporomandibular disorder (TMD).^30^ The primary goals of the OPPERA study were to identify putative psychological and physiological risk factors, clinical characteristics, and related genetic mechanisms that influence the development of chronic orofacial pain associated with TMD. In this prospective inception cohort study of 3263 individuals with no current or previous experience of TMD, the investigators collected a comprehensive battery of baseline and follow-up measures to predict those individuals who ultimately developed TMD. In addition to multiple psychosocial factors predicting development of TMD, the authors found 6 single-nucleotide polymorphisms as risk factors for chronic TMD.^4,92^ Through the Helping to End Addiction Long-term (HEAL) project, the NIH is currently initiating a multicenter trial to characterize risk factors and define susceptibility/risk biomarkers (including neuroimaging) to predict which individuals develop persistent pain after surgery. These initiatives and others aimed at identifying susceptibility/risk biomarkers may provide information that would allow for earlier identification of individuals at risk of developing persistent pain conditions and indicate modifiable factors (eg, diet, exercise, and behavioral change) that could be influenced to mitigate or reduce the susceptibility of chronic pain.

### 2.4. Predictive biomarkers

Predictive biomarkers are used to identify those individuals who are more likely to respond to a specific treatment.^7^ Biomarkers that can predict treatment responses are critical for the development and success of precision pain medicine approaches.^13^ Predictive biomarkers are typically used in pharmaceutical and device development to enrich a clinical study population for a subsequent randomized controlled trial (RCT).^6,87,99^ To date, most research in predictive biomarkers has been in the field of genetics for the prediction of cancer treatment response. For example, in a study of cancer treatments approved by the FDA from 1998 to 2013, researchers demonstrated that a biomarker-based approach to clinical trials of anticancer drugs was associated with improved efficacy and longer progression-free survival relative to conventional trials.^87^

Two examples from psychiatry illustrate the potential of neuroimaging-based predictive biomarkers to predict treatment responses. Amygdala reactivity and early life stress (ELS) both have been strongly implicated in the mechanisms of depression in animal and human models.^23,31,33^ Researchers integrated neuroimaging and ELS measures within a controlled trial of antidepressant outcomes.^33^ They demonstrated that the interaction between ELS and amygdala engagement predicted functional remission on antidepressants with more than 80% cross-validated accuracy. In depressed people exposed to high ELS, a greater likelihood of remission was predicted by amygdala hyperreactivity to socially rewarding stimuli, whereas for those with low-ELS exposure, amygdala hyporeactivity to both rewarding and threat-related stimuli predicted remission.^33^ Thus, amygdala reactivity and ELS are biobehavioral biomarkers for predicting functional remission in depression.

Like many pain conditions, depression is not a unitary disease; rather, it is a heterogeneous syndrome encompassing subtypes with varied, co-occurring symptoms and divergent treatment responses. Drysdale et al. used neuroimaging in a large multisite sample (n = 1188) to demonstrate that patients with depression can be divided into 4 neurophysiological subtypes defined by distinct patterns of dysfunctional connectivity in limbic and frontostriatal networks.^22^ The authors used this clustering to develop diagnostic biomarkers with high (82%–93%) sensitivity and specificity for depression subtypes in multisite validation (n = 711) and out-of-sample replication (n = 477) data sets. Interestingly, these subtypes could not be differentiated solely on the basis of clinical features but were associated with differing clinical-symptom profiles. The researchers used these biomarkers to predict responsiveness to transcranial magnetic stimulation therapy (n = 154), thereby identifying the individuals most likely to benefit from targeted neurostimulation therapies.^22^

Predictive biomarkers could help guide go/no-go decisions in selecting effective analgesics in early human drug development. Borsook et al. outlined the utility of functional imaging to define biomarkers to predict efficacy and safety, determine drug–dose relationships, and provide objective measure of symptom response and disease modification.^8–10^ A more recent example of this was by Wanigasekera et al.^107^ who assessed the utility of fMRI with a capsaicin-induced central sensitization (a mechanism relevant to neuropathic pain) to differentiate an effective (gabapentin) from ineffective (ibuprofen) treatment and both from placebo. They found that gabapentin reduced connectivity between the thalamus and secondary somatosensory cortex, whereas ibuprofen did not when compared with placebo. They also determined that the neural activity evoked by hyperalgesia from the right nucleus cuneiformis and the left posterior insula was more sensitive than the behavioral pain scores in detecting a statistically significant difference between gabapentin and placebo. This work built upon the group's previous work to generate and validate a general protocol for neuroimaging-based assessment of drug activity in the CNS that can be used to optimize drug discovery and validation.^23^

Predictive biomarkers would have obvious utility in the clinical field of pain medicine. Effect sizes for many analgesic efficacy RCTs in chronic pain are modest at best. Nonetheless, within each treatment group, there are often clear responders mixed with nonresponders.^13^ Neuroimaging-based biomarkers would be valuable in predicting these responders in an RCT.

The neuroimaging biomarker could be used either to select patients for participation or to stratify patients into biomarker-positive and biomarker-negative groups, with the primary endpoint being the effect in the biomarker-positive group. These biomarker enriched clinical trials could be used to identify effective treatments that might otherwise fail if more heterogeneous populations were enrolled.

Predictive pain biomarkers would also inform patient care decisions. Currently, clinicians are faced with a myriad, and increasing number, of treatment choices for pain including pharmacologic, psychological, interventional, physical therapy, complementary and alternative medicine, and self-management approaches. Unfortunately, outside of clinical experience, clinicians often have little information to guide them on treatment decisions. Predictive pain biomarkers could aid clinical decision-making in choosing the best treatment(s) for a specific patient with a specific pain condition under specific environmental circumstances, such as the role of long-term prescribing of opioids in chronic noncancer pain, which is one of the most important pain issues our country is facing.

According to the Centers for Disease Control and Prevention, opioid misuse, abuse, addiction, and associated overdose deaths have reached epidemic levels in the United States.^15^ More than 90 Americans die daily from opioid overdose.^93^ This health care crisis exists despite studies showing that long-term use is increasingly associated with significant negative side effects, risk of misuse, abuse, and addiction.^90,93,94^ However, stable doses of opioids may provide extended pain relief with limited side effects for a subgroup of individuals.^47^ Consequently, a poor risk/benefit ratio in a large patient population may obscure a positive profile in a subgroup of opioid-responsive chronic pain patients. Perhaps, one of the most valuable applications of predictive pain biomarkers would be in predicting those patients who would respond favorably, and with minimal adverse events, to opioids—and those who are at increased risk of misuse, abuse, diversion, and overdose.

The development of neuroimaging-based predictive biomarkers could transform our care of those in pain. From optimizing clinical trials to aiding clinical care decisions, predictive biomarkers are a critical component of realizing the goal of precision pain medicine.

### 2.5. Monitoring biomarkers

A monitoring biomarker is used to serially assess the presence, status, or extent of a medical condition, or to provide evidence of a treatment or adverse effect.^7^ This type of biomarker represents a change in a biomarker value across multiple points in time. As such, this biomarker category is broad and can include other types of biomarkers if they are assessed serially. For example, clinicians could use a neuroimaging monitoring pain biomarker to serially assess the progression of pain to persistent pain or opioid use after surgery or injury or the progression from chronic pain to high-impact chronic pain in an individual.^102^

### 2.6. Pharmacodynamic/response biomarkers

A pharmacodynamic/response biomarker is a biomarker whose levels change in response to an exposure to a medical product or an environmental agent. A change in a pharmacodynamic/response biomarker can provide evidence for clinical efficacy or assess an endpoint related to safety concerns. It can also provide clinical decision support for patient management to help determine whether to continue treatment or to adjust dose. In addition, these biomarkers can be useful for pharmaceutical/device development by assessing whether a treatment had a pharmacodynamic/device effect related to a clinical response. Because of the repeated nature of their assessment, pharmacodynamic/response biomarkers are also often considered *monitoring biomarkers*. For example, tricyclic antidepressants (TCAs) are a class of medications often used to treat chronic neuropathic pain. Clinicians can monitor blood levels of TCAs as a pharmacodynamic/response biomarker and use the results to titrate drug levels to within a therapeutic range.^78^ Similarly, the corrected QT interval is used as a safety biomarker to assess potential for drugs such as methadone or TCAs to induce torsades de pointes, a potentially fatal arrhythmia.

### 2.7. Safety biomarkers

Safety biomarkers detect or predict adverse drug or exposure effects.^7^ Many of the treatments used in pain management, particularly medications, have undesirable and potentially harmful or toxic effects. An example of this is the use of TCAs for treating neuropathic pain, as noted above. Although TCAs can be efficacious for pain, they can also have adverse effects (eg, drowsiness, constipation, cardiac arrhythmias and sudden death, blurred vision, and orthostatic hypotension). A neuroimaging-based safety biomarker would be useful if it could predict which treatments would negatively impact which patients under specific circumstances.

### 2.8. Need for multimodal biomarkers of pain

**Figure 2.**
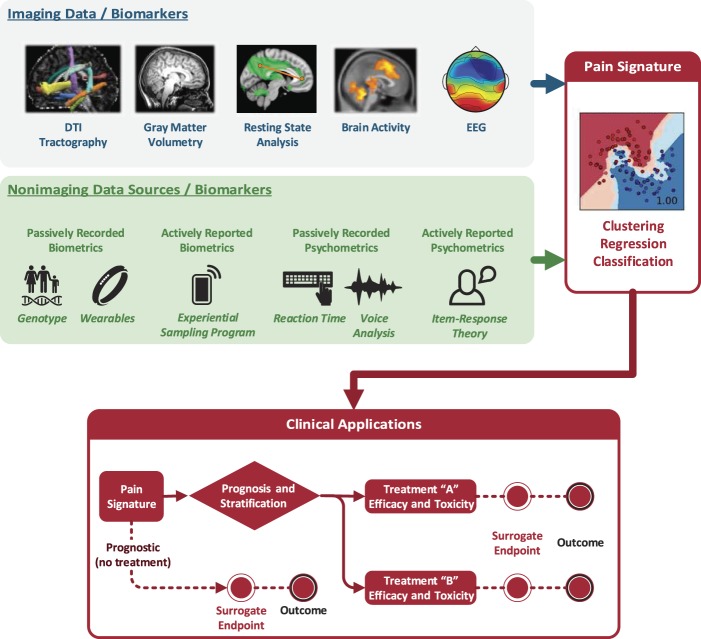
Multimodal Pain Signatures. Imaging data sources (top, gray box) and nonimaging data sources (middle, green box) can be combined into machine learning algorithms to provide a multimodal signature pattern of pain (right, red box). Various sources of imaging biomarkers include (1) structural changes measured with MRI (eg, diffusion tensor imaging [DTI] of white matter tractography; gray matter volumetry), (2) functional differences measured with fMRI (eg, resting state fMRI networks and functional connectivity between brain regions; brain activity in response to evoked stimulation or during a task), and (3) functional differences measured with non-MRI modalities such as EEG. Nonimaging data sources include, eg, genotype information, biometrics from wearable technology (eg, actigraphy), actively reported biometrics (eg, through handheld devices for recording patients' symptoms throughout the day), psychometrics including reaction time tests and voice analysis (eg, to measure emotional states such as depression or anxiety), and actively reported psychometrics (ie, demographic, psychological, and clinical questionnaires) (middle, green box). The multimodal pain signature can then be used in a variety of biomarker applications (bottom, red box). fMRI, functional MRI.

It is clear that neuroimaging alone will not capture all of the variance in models defining diagnostic, predictive, prognostic, and risk biomarkers. More likely, we will need to combine neuroimaging and non-neuroimaging data (eg, behavioral, genotype, phenotype, and longitudinal data) into a multimodal biomarker of pain. Combining measures of genomics and other 'omics, activity monitors, passively recorded psychometrics, and quantitative sensory testing with neuroimaging data could improve the sensitivity and specificity of neuroimaging-based biomarkers. Figure 2 illustrates the integration of multiple potential biomarkers and provides a schematic of how these different types of biomarkers may be applied.

## 3. A framework for evaluating neuroimaging-based biomarkers of pain

Neuroimaging-based pain biomarkers must be validated to have true clinical and research utility. Validation is “a process to establish that the performance of a test, tool, or instrument is acceptable for its intended purpose.”^7^ It is critical to confirm that a neuroimaging pain biomarker measures what it is intended to measure and that it predicts or measures the relevant clinical concept (ie, it has appropriate analytical and clinical validity). A biomarker must also have clinical utility, in that it provides information that assists in the care of patients. An essential first step in evaluating a neuroimaging-based pain biomarker is to precisely define the brain measures it is intended to assay, the pain condition of interest, the purpose of the test, and the population or health care setting in which it is going to be used. In other words, context is paramount in considering the validity and utility of a neuroimaging-based pain biomarker to achieve clinical or research utility.

**Figure 3.**
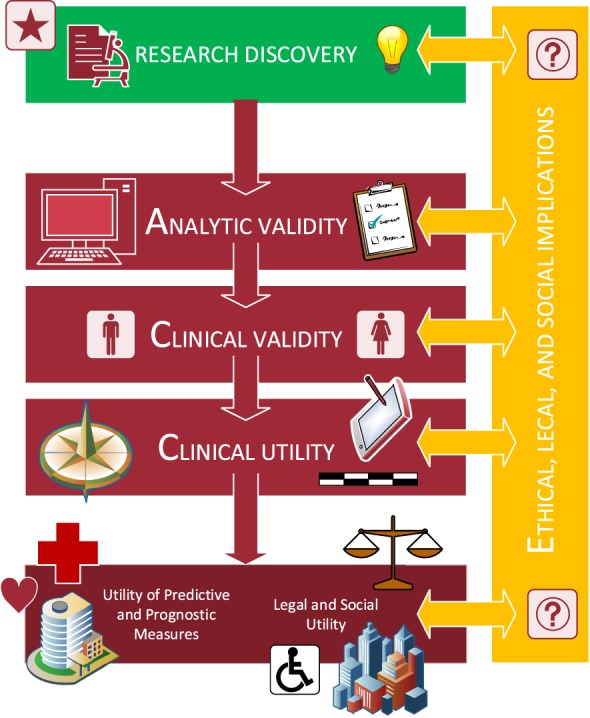
Framework for Evaluating Neuroimaging-based Biomarkers of Pain. A candidate biomarker will need to be vetted through all stages of analytic validity, clinical validity, clinical utility, and ethical, legal, and social implications.

To formally evaluate a biomarker, we can draw upon other fields that have developed evaluation frameworks. One such framework that researchers have successfully adopted in the field of genetic testing is the ACCE criteria.^71,88,115^ The ACCE framework was established by the Centers for Disease Control Prevention Office of Public Health Genomics to set a standard path for genetic tests to follow toward clinical and public use.^55^ After initial research discovery, the framework includes the ACCE acronym stages of (1) Analytic Validity (2) Clinical Validity (3) Clinical Utility, and (4) Ethical, Legal, and Social Impacts^37,115^ (Fig. 3). The ACCE model includes a standard set of 44 questions to ensure proper application and to safeguard against critical social, ethical, and legal issues.^37^ Although originally written for the purpose of diagnostic genetic tests, these questions can be adapted to other types of biomarkers listed in Section 2. For example, we have adapted these standard questions to reflect an approach for diagnostic neuroimaging pain biomarkers.

The National Academies of Medicine (NAM) recently completed a report entitled “An Evidence Framework for Genetic Testing.”^1^ In this report, the authors built upon ACCE and other testing frameworks to refine the notion of analytic validity, clinical validity, and clinical utility and emphasize the importance of integrating societal benefit into the evaluation process.^1^

In 2010, a landmark report from NAM provided a foundation for evaluating biomarkers and surrogate endpoints.^89,104^ This report recommended an evaluation process that included: “(1) Analytical validation: analyses of available evidence on the analytical performance of an assay; (2) Qualification (NB: Qualification as used here is synonymous with clinical utility used in ACCE): assessment of available evidence on associations between the biomarker and disease states, including data showing effects of interventions on both the biomarker and clinical outcomes; and (3) Utilization: contextual analysis based on the specific use proposed and the applicability of available evidence to this use. This includes a determination of whether the validation and qualification conducted provide sufficient support for the use proposed.”^89,104^ These steps are interrelated and not separated in time. Therefore, conclusions in 1 step may require revisions in other steps.

The FDA Center for Drug Evaluation and Research Biomarker Qualification Program's mission is to work with external stakeholders to develop biomarkers as drug development tools.^27^ Their program outlines a multistep process for a biomarker to be qualified for drug development. This program relies heavily on the BEST resource outlined above as a “living” glossary of terms used in biomarker qualification science and medical product development. The FDA's program relies heavily on defining a specific context of use for the biomarker to be used in drug development. The FDA defines the Context of Use as *a complete and precise statement that describes the appropriate use of the biomarker and how the qualified biomarker is applied in drug development and regulatory review.* The context of use statement also describes important criteria regarding the circumstances under which the biomarker is qualified. As with the other frameworks above, the FDA Biomarker Qualification Program involves both analytical validation and clinical validation for approval. Here, the FDA defines analytical validation as to “establish that the preanalytical considerations and performance characteristics acceptably support the biomarker's context of use,” while clinical validation is to “establish that the biomarker acceptably identifies, measures, or predicts the concept of interest.” Two important points about the FDA Biomarker Qualification Program is in what it does not do. Specifically, the program does not imply the test/assay has been reviewed by the FDA and cleared or approved for use in patient care. In addition, the qualification does not qualify the biomarker for use in clinical practice.

In summary, there is more commonality with these frameworks than differences. They all require analytical and clinical validity. The differences are more to do with the intended purposes of the framework, with for instance, the FDA Biomarker Qualification Program aimed more at drug development and the others having a broader utility.

### 3.1. Analytical validity

The analytical validity of a neuroimaging-based biomarker refers to “assessing [an] assay and its measurement performance characteristics and determining the range of conditions under which the assay will give reproducible and accurate data.”^105^ In this context, an *assay* is a method to analyze or quantify brain activity or a structure in an individual or animal. Analytical validity is concerned with assessing the performance of a biomarker test in a laboratory setting as opposed to the clinic or general population. Here, we need quality assurance to ensure that the results are reliable and reproducible across scanners, clinical settings, and analysis pipelines.

Pooling of neuroimaging data across centers and scanners will be required to yield the numbers of subjects to demonstrate proper analytical and clinical validity. Multiple neuroimaging research networks have addressed the challenges of pooling multicenter neuroimaging data, as well as developing common informatics, quality control, analysis, and visualization tools and protocols. These networks include the International Consortium for Brain Mapping,^68^ Alzheimer' Disease Neuroimaging Initiative (ADNI),^44^ Function Biomedical Informatics Research Network (fBIRN),^32^ National Institutes of Health Pediatric MRI Database,^24^ and the Human Connectome Project.^60,101^ As part of the National Institutes of Health-funded Multi-Disciplinary Approach to the Study of Chronic Pelvic Pain (MAPP), we emulated many of these pioneering research networks in establishing the neuroimaging protocols, databases, analyses, and visualizations used across 5 research centers with different manufacturers and models of MRI scanners. Alger et al. outlined many of the challenges and solutions to neuroimaging across multiple centers and scanners within the MAPP network.^2^ The MAPP network subsequently published multiple articles demonstrating the feasibility and insights that are possible with large multicenter neuroimaging data sets of pain.^3,28,43,48,50–52,54,65,66,96,97,113^

The analysis approach and algorithms used in analytical validation are critical. Most approaches are using a multivariate pattern analysis or machine learning approach. An important factor in the context use of such analyses is the embedding of feature selection, classifier optimization, and the estimation of the models' generalizability in a cross-validation scheme. In the cross-validation steps, it is important to avoid information leakage between the training and the test samples to avoid overfitting and inflated estimates of classification accuracy. Moreover, even in the case of correct feature embedding, different cross-validation schemes might lead to different results. In a multivariate pattern analysis meta-analysis for detecting neuroimaging biomarkers of depression, the authors identified that 2-fold cross-validation was associated with higher diagnostic accuracy than 10-fold or leave-one-out cross-validation.^45^ We will need generalizability of the models across research centers as well as agreement on the identification of the methodological and clinical variables moderating classification success.

The biomarker must also show adequate sensitivity and specificity before it is assessed in subsequent biomarker evaluation steps. This quality assurance will typically include both internal and external control assessments within a structured framework. Analytical validation can also include determining the extent to which data from different tests for the same biomarker may be compared to one another. Highly comparable data strengthen the biomarker and add power to retrospective analyses of data related to the biomarker.^89^

### 3.2. Clinical validity

Clinical validity is defined as the “evidentiary process of linking a biomarker with biological processes and clinical endpoints.”^89^ Clinical validity (1) defines the ability of a neuroimaging pain biomarker to detect or predict the presence or absence of a phenotype or clinical disease or (2) defines a biomarker's ability to predict the effects of interventions on clinical endpoints of interest. If a biomarker-clinical endpoint relationship occurs over several interventions, we can consider the biomarker more generalizable. Candidate biomarkers that are both informative of pathophysiology and highly prognostic (ie, able to identify risk factors for developing disorders and disease predisposition) should pass standards of diagnosticity, interpretability, deployability, and generalizability. Similarly, candidate biomarkers will need to differentiate CNS features associated with different clinical pain conditions—assuming such features exist. Are the CNS features of chronic low back pain the same or different as compared with CRPS, migraine, pelvic pain, or fibromyalgia? Are there biomarker differences in a painful condition that is presumed to be more peripherally vs centrally driven? As noted previously, in a cohort of localized chronic pelvic pain vs pelvic pain plus widespread pain, we identified different neuroimaging biomarkers that distinguish both groups.^50^ Assuming there are differences in features amongst painful conditions, do they matter regarding prognosis of natural history or prediction of treatment response? In addition, candidate biomarkers will need to account for patient heterogeneity introduced with age, severity and duration of pain, concomitant medications or other treatments, and comorbid conditions such as depression, anxiety, and catastrophizing. In addition, population base rates will need to account for prevalence of chronic pain syndromes,^69,83^ similar to previous applications for genetic and psychological testing. Formally evaluating the sensitivity, specificity, positive predictive value, and negative predictive value of the biomarker is also pertinent. Researchers must include appropriately selected controls in any formal biomarker evaluation.

### 3.3. Utilization or clinical utility

Utilization refers to the “contextual analysis based on the specific use proposed and the applicability of available evidence to this use. This includes a determination of whether the validation and qualification conducted provide sufficient support for the use proposed.”^89^ Clinical utility assesses the likelihood that the biomarker will lead to an improved clinical outcome. Clinical utility helps address the purpose of the biomarker? Do the biomarker findings change clinical management or prognosis? What is the natural history of the disorder? Are there effective interventions based on the biomarker results? Is the biomarker result information useful for family members? Does the biomarker give rise to any ethical, legal, or social consequences? Are there other ways of achieving the same purpose apart from using a neuroimaging biomarker? For example, and as mentioned previously, there is little clinical utility for a neuroimaging pain biomarker to simply determine in a binary manner if a person seen in a clinical setting is in pain or not. We can simply just ask. What is the cost of the neuroimaging biomarker? Is the biomarker cost effective? In these latter 2 questions on cost, we will have to consider that at $500+/hour of scanner time, neuroimaging biomarkers are expensive. For broad use, more cost-effective MRI systems will need to be developed or other imaging modalities used (eg, EEG and functional-near infrared spectroscopy) that are less costly.

### 3.4. Ethical, legal, and social implications

The NAM and ACCE frameworks formally consider ethical, legal, and social implications of biomarkers. This component of the biomarker evaluation is perhaps the most difficult to address as it is wide ranging.

A neuroimaging biomarker that accurately detects pain could be useful in society for reasons beyond improving patient care. For example, in the legal system, the existence and extent of pain cannot be taken for granted but is at the heart of a dispute. In many personal injury cases, plaintiffs seek damages for ongoing pain that may have little evidence beyond self-report. Even more often—hundreds of thousands of times a year—in disability determinations, workers claim to private disability insurers or to Social Security that pain makes them incapable of working. The courts and administrative agencies cannot always by default accept the claimant's self-report as true, but often, there is little other evidence. While clinicians and researchers usually can “just ask” patients if they are in pain, the incentives involved means the legal system cannot blindly trust their answers, particularly in the American adversary system where there is always “the other side” fighting against any claims. Not only can this lead to people being granted or denied damages or benefits improperly but also the uncertainty involved increases the time and money spent in litigation.^46,80^ Further legal applications of neuroimaging-based pain biomarkers are beyond the scope of this review, but have been described in the following reviews.^19,81^

We must also consider several legal concerns regarding pain biomarkers.^34^ First, what would be necessary before neuroimaging evidence of pain could be accepted into evidence to use in a court or an administrative proceeding? A neuroimaging-based biomarker would have to perform at an acceptable level of accuracy in at least 3 areas: (1) accuracy with respect to demographics and phenotype (eg, men vs women, young vs elderly, mentally ill, and illegal drug users), (2) accuracy with different kinds of pain (eg, acute vs chronic, low back pain, and migraine), and (3) accuracy that cannot be undermined by countermeasures to “fool” the neuroimaging-based biomarker.

In addition to these issues, we must resolve the deeper question of “how accurate would it need to be?” The law does not set standards based on *P* values or confidence intervals; judges will need to decide whether it is “sufficiently” reliable to help the legal process.^36^ Second, even if a neuroimaging-based biomarker met the vague standards for accuracy, could a claimant be forced to undergo such a test? Typically, people can be forced to undergo medical examinations at the request of their legal opponents or forfeit their claims. Is it different when the test involves probing an individual's interior mental states vs a vertebral disk? The question whether there is, or should be, some kind of “cognitive liberty” that is free from compulsory intrusion is important, yet undecided.^25,26^ Although a reliable brain biomarker of pain would clearly be of benefit to the legal system, the additional requirements for fair and just use are complicated, and misguided use could profoundly impact patients' lives beyond their clinical care and outcomes.

Perhaps, the greatest risk may be the premature availability of such tests and their use by patients, doctors, employers, agencies, or judges when they have not been shown to be “accurate enough.” Our system has incentives for individuals to use any evidence they can to try to persuade others, as well as incentives to lead some people to sell goods and services to others without knowing they are effective—and, in the cases of some frauds, while consciously knowing that they are ineffective.

Before and during each of the above evaluation stages, critical questions must be addressed. For example, we must consider the impact of the biomarker on insurance and employment, health care disparities including equity and access, privacy and confidentiality, and stigmatization. As alluded to earlier, there is great potential for misuse and abuse of these neuroimaging-based pain biomarkers with real potential for stigma and discrimination. An early example of this involved broad-based community screening efforts for sickle cell disease in the 1970s. These screening efforts were accompanied by a misunderstanding of the health implications of the carrier state, leading to subsequent insurance and employment discrimination.^106^ This, and other discrimination events around genetic testing, led to the federal Genetic Information Nondiscrimination Act in 2008, which blocks health insurers and employers from using genetic information (but not, eg, neuroimaging information) in health coverage or employment decisions. The Patient Protection and Affordable Care Act of 2010 added further protections, although their continuation is in some doubt. Nonetheless, in any event, there is still potential for discrimination not covered under these acts including determination of life insurance, mortgage insurance, long-term care insurance, and long-term disability insurance. Importantly, there are few protections in place for the misuse of neuroimaging-based pain biomarkers. As such, these potential issues will require scientists, clinicians, ethicists, attorneys, and patients to be vigilant for such misuse and abuse.

## 4. Conclusions

We have reviewed the different types of potential neuroimaging-based pain biomarkers, their clinical and research applications, and their limitations. The field of neuroimaging-based biomarkers has advanced rapidly. With this rapid advancement comes a need for structured frameworks and processes to validate them as biomarkers. We have presented such a framework adapted from several successfully applied frameworks in other fields. This model of assuring analytical validity, clinical validity, and clinical utility and accounting for ethical, legal, and social implications can help advance these biomarkers to achieve broad clinical and research utility while minimizing the risks of misapplication of this emerging technology. Neuroimaging pain biomarkers are helping to advance the goal of personalized pain medicine to ultimately aid clinicians and patients to choose the best treatment that both safe and effective.

## Disclosures

The authors have no conflict of interest to declare.

This work was supported by NIH grants DA029262 (K24, SCM), DA040154 (K99, KTM), and GM089626 (T32) and the Redlich Pain Research Endowment.
